# Supplementary material for: MetaRibo-Seq measures translation in microbiomes
Source: Nat Commun. 2020 Jun 29;11:3268. doi: 10.1038/s41467-020-17081-z (PMC7324362; doi:10.1038/s41467-020-17081-z)
Supplement: Supplementary file 10 — Supplementary Data 7 [file 41467_2020_17081_MOESM10_ESM.zip › File2/Confidence_High_Taxonomy/308939_out.krona.html]

Javascript must be enabled to view this page.

members
magnitude
magnitudeUnassigned
count
unassigned
taxon
rank

308939\_out

3

2
superkingdom
3

3
phylum
1239


SRS104400\_contig\_number\_54982
species
1897027
1

186801
class
2

2
order
186802

1950913
species

SRS016203\_contig\_number\_11651SRS063985\_contig\_number\_29004
2
